# Evaluating Early Macrolide Therapy in Pediatric Campylobacter Enterocolitis: A Comparative Study

**DOI:** 10.3390/antibiotics15020171

**Published:** 2026-02-05

**Authors:** Ho Jung Choi, Yoon Kyung Cho, Ye Ji Kim, Hyun Mi Kang, Dae Chul Jeong, In Hyuk Yoo

**Affiliations:** 1 Department of Pediatrics, College of Medicine, The Catholic University of Korea, Seoul 06591, Republic of Korea; fisherhj@naver.com (H.J.C.); dcjeong@catholic.ac.kr (D.C.J.); 2 Vaccine Bio Research Institute, College of Medicine, The Catholic University of Korea, Seoul 06591, Republic of Korea

**Keywords:** *Campylobacter* enterocolitis, macrolide, pediatric, polymerase chain reaction (PCR) tests

## Abstract

**Background/Objectives:** Azithromycin is widely recommended as the first-line treatment for pediatric *Campylobacter* enterocolitis, although supporting evidence is limited and there is a lack of studies evaluating the efficacy of other macrolide antibiotics. This study aims to assess the effectiveness of starting macrolide therapy within three days of symptom onset in pediatric patients with *Campylobacter* enterocolitis. **Methods:** Pediatric patients under 19 years of age with a new diagnosis of *Campylobacter* enterocolitis were enrolled and randomly assigned to receive macrolide antibiotic treatment with either azithromycin or clarithromycin in a 1:1 ratio. Additionally, a retrospective historical cohort of pediatric patients diagnosed with *Campylobacter* enterocolitis prior to the study period, who did not receive macrolide antibiotics, was retrospectively reviewed for comparison. This dual approach allowed for the evaluation of macrolide therapy’s effectiveness against untreated cases. **Results:** The study included 27 patients in the macrolide group and 37 patients in the non-macrolide group. Baseline demographic and clinical characteristics were comparable between groups. Early macrolide therapy was associated with reduced hospital stay (3.8 ± 0.7 vs. 4.5 ± 0.9 days), shorter duration of diarrhea (1.8 ± 1.2 vs. 3.4 ± 0.7 days, *p* < 0.001), and shorter duration of fever (1.1 ± 0.6 vs. 2.8 ± 1.0 days, *p* < 0.001). No significant difference was observed in the duration of vomiting (*p* = 0.061). **Conclusions:** Early initiation of macrolide antibiotics in children with *Campylobacter* enterocolitis significantly accelerated complete clinical resolution and shortened hospitalization, particularly by hastening the resolution of diarrhea, fever, and abdominal pain. These findings support the use of early macrolide therapy for pediatric *Campylobacter* enterocolitis.

## 1. Introduction

Acute gastroenteritis (AGE) is one of the most common illnesses in children, and in otherwise healthy patients, antibiotic therapy is usually unnecessary [1,2]. This is primarily because the disease is generally self-limiting, with symptoms resolving spontaneously in most cases, pathogens being eliminated within days to weeks, and complications remaining rare [3,4].

Even in bacterial AGE, empiric antibiotic therapy is not routinely indicated. However, when a specific pathogen that responds to antibiotics is identified, treatment may be initiated accordingly. In cases where the pathogen is not confirmed, empiric therapy can be considered for patients with severe disease, those who are very young, or individuals with significant underlying comorbidities [1,2]. In the past, antibiotics were often prescribed empirically because conventional stool culture or polymerase chain reaction (PCR) testing took several days for pathogen identification. However, with the advent of rapid syndromic multiplex panels, real-time pathogen-based management has become increasingly feasible [5,6,7,8].

Among bacterial pathogens, *Campylobacter* species are distinctive because antibiotic therapy is more strongly recommended for them than for many other causes of acute gastroenteritis (AGE) once they are identified [9,10]. In pediatric *Campylobacter* enterocolitis, macrolide antibiotics are recommended for patients experiencing dysenteric symptoms, including bloody or mucoid diarrhea, high fever, severe abdominal pain, or tenesmus. These antibiotics are also advised for public health reasons to reduce transmission in institutional settings [2,11].

The clinical benefit is most apparent when antibiotics are started within the first three days of illness [12]. *Campylobacter* species are intrinsically resistant to several classes of antibiotics, including penicillins and cephalosporins, making macrolides the preferred treatment [13,14]. Historically, erythromycin was considered the first-line treatment [15]; however, azithromycin has since become the preferred option for children because of its favorable safety and pharmacologic profile. Current pediatric guidelines now endorse azithromycin as the standard therapy [2,16].

However, current recommendations are primarily based on a meta-analysis of outdated studies conducted in the 1980s and 1990s, primarily involving adults, which creates a contemporary evidence gap in pediatric practice, especially in the era of rapid diagnostic testing [12]. These studies were small in scale and included only a limited number of pediatric patients treated with erythromycin, which is no longer the preferred antibiotic for children. As a result, the evidence base for current guidelines is outdated and does not fully represent contemporary pediatric practice. Since then, only one pediatric trial has assessed the benefits of antibiotic therapy in *Campylobacter* enterocolitis, and little additional evidence has been generated [17].

Given that *Campylobacter* enterocolitis is one of the most common bacterial diarrheal diseases in children [9], there is a need for updated data on the clinical effectiveness of antibiotics—especially in the current era of rapid diagnostic testing, which enables timely pathogen-directed treatment.

The present study aimed to assess the clinical benefits of early macrolide therapy in pediatric *Campylobacter* enterocolitis. We evaluated children who presented within three days of symptom onset, comparing those treated with macrolides to those who either received no antibiotics or were given antibiotics to which *Campylobacter* species are inherently resistant [18]. We aimed to assess whether early macrolide therapy offers measurable clinical benefits for this common pediatric infection.

## 2. Results

### 2.1. Baseline Characteristics

This study included 64 pediatric patients diagnosed with *Campylobacter* enteritis. Among the 27 patients enrolled in the macrolide group, 14 received azithromycin and 13 received clarithromycin. The non-macrolide group comprised 37 patients who did not receive macrolides; instead, they were treated empirically with non-macrolide antibiotics for suspected bacterial acute gastroenteritis while awaiting PCR or stool culture results. In this group, initial empiric antibiotics were administered at a median of 51 h from symptom onset. One patient received amoxicillin/clavulanate, two received cefuroxime, and 34 received intravenous cefotaxime. Since these agents were used empirically before pathogen identification and *Campylobacter* is intrinsically resistant to them, the timing of their administration was considered to have minimal impact on clinical resolution.

Baseline demographic, clinical, and laboratory characteristics were similar between the two groups (Table 1). There were no significant differences in mean age, sex, or duration of illness prior to treatment initiation. Laboratory findings—including WBC, hemoglobin (Hb) levels, platelet count, CRP, BUN, creatinine, AST, ALT, total bilirubin, and electrolytes—also showed no significant differences between groups, except for a lower mean sodium level in the macrolide group (*p* < 0.001). The presence of acute enterocolitis symptoms at presentation (diarrhea (≥4 times/day), fever, bloody stool, vomiting, and abdominal pain) did not significantly differ between the two groups.

### 2.2. Comparison of Primary Outcome

**Figure 1:**
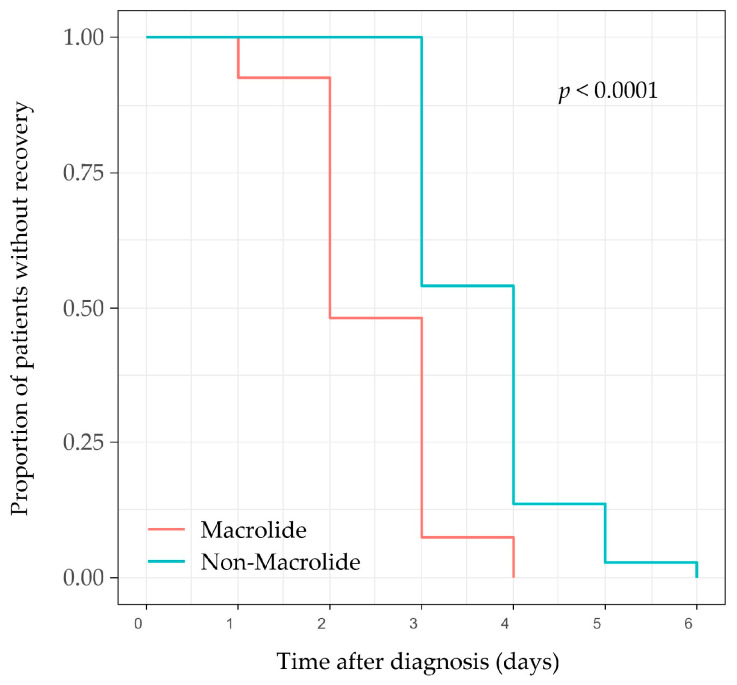
Kaplan–Meier Curve for time to complete clinical resolution.

Early macrolide therapy resulted in a statistically significant earlier complete resolution of clinical symptoms compared to non-macrolide therapy (Table 2, Figure 1). In the macrolide group, the median time to complete clinical resolution was 2 days (IQR 2–3), while in the non-macrolide group it was 4 days (IQR 3–4) (log-rank *p* < 0.001). Cox proportional-hazards regression analysis further confirmed that early macrolide therapy was independently associated with a shorter time to complete clinical resolution (adjusted HR = 2.66; 95% CI, 1.44–4.93; *p* = 0.002; Table 3). Although serum sodium was included as a covariate due to its baseline difference, the Variance Inflation Factor (VIF) for all variables in the model was below 1.5, confirming the absence of significant multicollinearity and the stability of the adjusted estimates.

Kaplan–Meier survival curves comparing the cumulative incidence of complete clinical recovery between patients who received early macrolide therapy (within 3 days of symptom onset) and those who did not show that patients treated with macrolides experienced a significantly shorter time to complete clinical resolution compared to the non-macrolide group (log-rank test, *p* < 0.001). The shaded areas represent the 95% confidence interval.

### 2.3. Comparison of Secondary Outcomes

**Figure 2:**
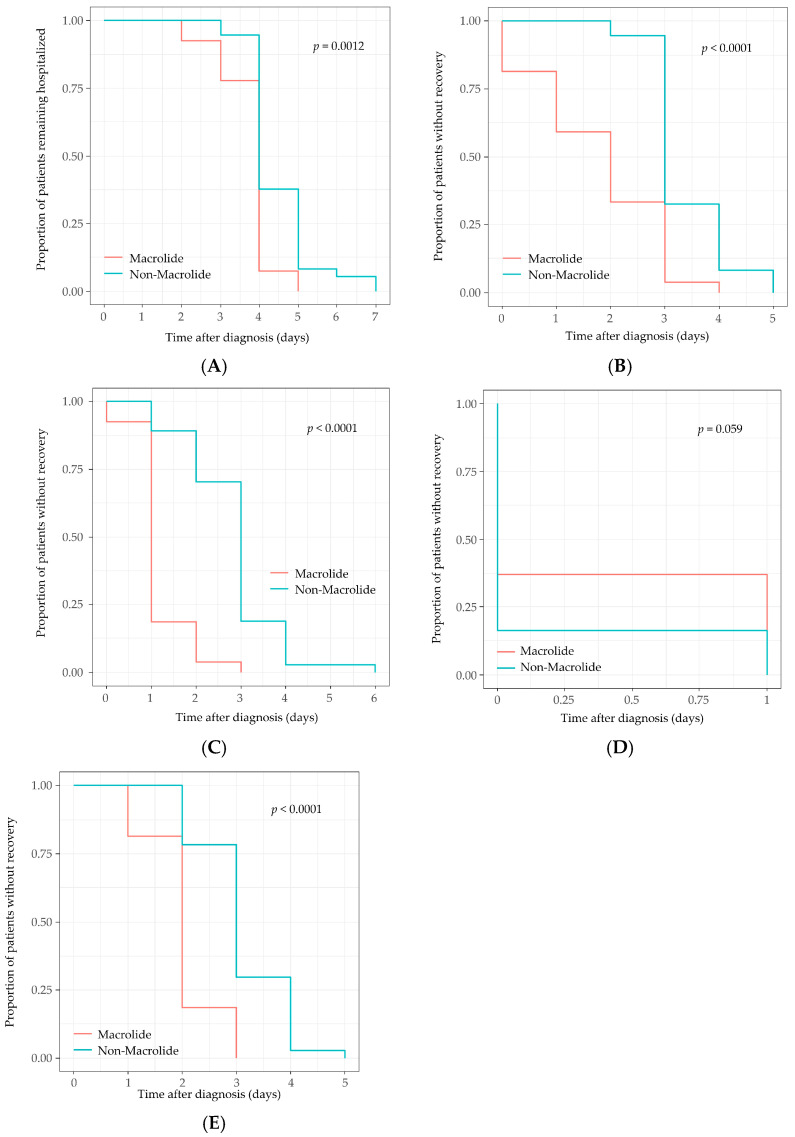
Kaplan–Meier Curves for time to resolution of individual clinical symptoms. Cumulative incidence plots depicting time to improvement of (**A**) Length of hospitalization, (**B**) Diarrhea, (**C**) Fever, (**D**) Vomiting, and (**E**) Abdominal pain.

The length of hospitalization and the time to resolution of diarrhea, fever, and abdominal pain were significantly shorter in patients receiving macrolide therapy (Table 2, Figure 2).

Children in the macrolide group were discharged a median of 1 day earlier than those in the control group, with a mean hospital stay of 3.8 ± 0.7 days compared to 4.5 ± 0.9 days (*p* = 0.002), corresponding to an adjusted hazard ratio (HR) of 1.69 (95% CI, 1.00–2.86; *p* = 0.049). The time to improvement in diarrhea (defined as fewer than 4 episodes per day) was shorter by approximately 1.5 days on average (1.8 ± 1.2 vs. 3.4 ± 0.7 days; *p* < 0.001), with an adjusted HR of 2.82 (95% CI, 1.66–4.78; *p* < 0.001). Fever resolution occurred more rapidly in the macrolide group (1.1 ± 0.6 vs. 2.8 ± 1.0 days; *p* < 0.001), with a fourfold higher daily probability of defervescence (adjusted HR = 4.50; 95% CI, 2.42–8.36; *p* < 0.001). The duration of vomiting did not differ significantly between the groups (adjusted HR, 0.81; *p* = 0.409) (Table 4).

### 2.4. Sensitivity Analyses

Sensitivity analyses using both the unadjusted (Model 1) and adjusted (Model 2) Cox models yielded consistent effect estimates, reinforcing the robustness of the findings (Table 3 and Table 4).

## 3. Discussion

This study evaluated whether early antibiotic therapy provides measurable clinical benefits for children with *Campylobacter* enterocolitis and found significant advantages. Initiating treatment within 72 h of symptom onset led to a shorter time to complete resolution of gastroenteritis-related symptoms. Diarrhea, fever, and abdominal pain resolved more quickly, and the daily probability of recovery was higher in the early-antibiotic (macrolide-based) group. Treatment also reduced the length of hospitalization by a median of one day. This rapid improvement is not only statistically significant but also clinically substantial in the pediatric context, as it significantly hastens the child’s return to baseline health status and reduces the period of acute distress. Furthermore, the observed reduction in hospitalization—while modest in days—represents a meaningful decrease in healthcare utilization and may alleviate the emotional and economic burden on caregivers. While a trend toward improvement was noted in vomiting, the results did not reach statistical significance (*p* = 0.061), suggesting that these areas require further investigation in larger, dedicated trials.

These findings indicate that timely, pathogen-directed antibiotics accelerate clinical recovery in pediatric *Campylobacter* enterocolitis. Our study highlights the clinical superiority of early pathogen-directed macrolide treatment over initial empiric antibiotic use, rather than comparing antibiotic treatment versus no treatment.

Our findings align with prior evidence. Meta-analytic data indicate that early antibiotics can shorten diarrhea by approximately 1 to 1.5 days and may reduce fecal *Campylobacter* shedding. The benefits are most pronounced when treatment begins within three days of symptom onset [12]. In a randomized pediatric trial, a single 30 mg/kg dose of azithromycin accelerated clinical cure compared to erythromycin or no antibiotic, highlighting the time-sensitive benefits of pathogen-directed therapy [17]. In line with these data, our study confirmed a significantly shorter time to complete clinical resolution and faster improvement in diarrhea, fever and abdominal pain with early antibiotics. Notably, the two-day reduction in the time to achieve complete clinical resolution (median 2 vs. 4 days) highlights the robust clinical benefit of early pathogen-directed therapy in accelerating the recovery of pediatric patients. Importantly, we also demonstrated a reduction in length of stay, a clinically meaningful benefit that has not been consistently observed in previous pediatric studies.

Historically, the impact of targeted therapy for acute bacterial gastroenteritis was limited due to delays in pathogen identification, as stool cultures or conventional PCR tests typically took several days [11]. With the advent of rapid syndromic multiplex panels, pathogen confirmation can now be achieved within hours for selected patients. This creates a realistic opportunity for early, organism-directed antibiotic therapy in severe or high-risk cases [5,7,19]. In this context, our findings offer timely and clinically significant evidence that supports early antibiotic treatment in appropriately selected pediatric patients.

It is important to note that all patients in the present study required hospitalization due to the severity of their illness, reflecting a population with clinically significant disease warranting antibiotic consideration. Current guidelines recommend antibiotic therapy in pediatric *Campylobacter* enterocolitis for patients with persistent high fever, prolonged bloody diarrhea, severe abdominal pain, or significant dehydration, as well as for high-risk groups such as young infants, immunocompromised patients, or those with signs of systemic infection. Administering antibiotics to patients who do not meet these clinical criteria may contribute significantly to the development of antimicrobial resistance without providing substantial clinical benefit.

Antibiotic stewardship is essential. In otherwise healthy children, most acute gastroenteritis cases are self-limiting, and indiscriminate antibiotic use poses risks, including adverse events and the development of resistance. Furthermore, for certain pathogens, antibiotics may actually worsen outcomes [20]. In contrast, when the causative organism is identified and is known to respond positively to antibiotics, and when the severity of the illness justifies intervention, targeted therapy can offer significant clinical benefits [3]. In such settings, appropriate antibiotic use can accelerate the resolution of clinical symptoms, reduce the risk of complications, and limit the transmission of infection. In our cohort, no adverse events related to antibiotic administration were reported. Although safety outcomes were not systematically analyzed, the lack of observed treatment-related adverse events supports the tolerability of early antibiotics in this pediatric population.

This study has several limitations. First, the retrospective nature of the non-macrolide control cohort may have introduced selection and information bias. The single-center design and heterogeneity of empiric agents in the control group could also confound our estimates. Additionally, several constraints must be acknowledged, including the lack of a systematic and formal assessment of treatment-related adverse events, the omission of post-therapeutic microbiological evaluations to confirm pathogen clearance, and a constrained sample size that potentially limits the statistical power to identify less pronounced effects or rare clinical events. Furthermore, while this study identified the clinical impact of pathogen-directed macrolide therapy on the duration of hospitalization, it should be noted that the duration of hospitalization may be influenced by institutional clinical practices and discharge protocols rather than clinical recovery alone. It should be regarded as a hypothesis-generating result that warrants validation in multicenter prospective studies. However, the study also has strengths: it featured prospective enrollment and randomized allocation in the macrolide arm, treatment initiation within 72 h, inclusion of hospitalized children with clinically severe illness, and balanced baseline characteristics. Prespecified adjusted and sensitivity analyses yielded consistent findings, further supporting the robustness of the results.

Future studies should include fully prospective, multicenter trials comparing early antibiotics with standardized supportive care. These trials should incorporate microbiologic endpoints, such as time to culture/PCR negativity, and monitor resistance patterns and post-infectious sequelae. Evaluating outpatient applicability and cost-effectiveness in the era of rapid diagnostics will further inform clinical and policy decisions. Collectively, these lines of inquiry could help translate early, pathogen-directed therapy into streamlined care pathways that improve outcomes and optimize healthcare utilization.

## 4. Materials and Methods

### 4.1. Study Design and Participants

This study was a single-center, comparative cohort analysis with both prospective and retrospective elements, conducted at a tertiary pediatric medical center. Its objective was to evaluate the clinical efficacy of early macrolide therapy in children diagnosed with *Campylobacter* enterocolitis. Data for the macrolide group were collected prospectively from July 2022 to October 2023, while data for the non-macrolide group were retrospectively obtained from electronic medical records spanning July 2015 to February 2022. To minimize potential information bias, the clinical evaluators responsible for data extraction and outcome assessment were blinded to the specific treatment timing during the initial evaluation process, although treatment was not assigned by the researchers due to the observational nature of the study.

Patients in the macrolide group received either azithromycin (10 mg/kg once daily for 3 days) or clarithromycin (7.5 mg/kg twice daily for 5 days), with treatment initiated within 3 days of symptom onset. Allocation between azithromycin and clarithromycin was randomized in a 1:1 ratio. The inclusion of both azithromycin and clarithromycin in this study reflects current real-world clinical practice and the availability of therapeutic options at our institution. The non-macrolide control group, which did not receive macrolides, was also analyzed retrospectively from electronic medical records. All patients were hospitalized with suspected bacterial enterocolitis and were empirically treated with non-macrolide antibiotics such as cefotaxime or amoxicillin-clavulanate while awaiting PCR or stool culture results.

The study protocol received approval from the Institutional Review Board of Seoul St. Mary’s Hospital (KC25RISI0102) and was conducted in accordance with the Declaration of Helsinki. Written informed consent was obtained from the legal guardians of all participants enrolled prospectively.

### 4.2. Inclusion and Exclusion Criteria

Patients aged 18 years or younger were eligible for inclusion if they had a confirmed *Campylobacter* infection, as verified by laboratory testing, and displayed clinical symptoms consistent with acute enterocolitis—such as diarrhea, fever, vomiting, bloody stool, or abdominal pain—within 72 h of symptom onset. Laboratory confirmation of *Campylobacter* infection was defined by the detection of *Campylobacter* species in stool samples, either through stool culture or culture-independent diagnostic tests (e.g., PCR). All patients required hospitalization due to the severity of their symptoms and presented with laboratory evidence of bacterial infection in their initial stool and blood samples. They were monitored daily for the frequency of diarrhea, fever, vomiting, and abdominal pain.

To ensure that any treatment effect observed could be attributed to the intervention, patients were excluded if they had taken antibiotics in the previous 7 days, had a chronic gastrointestinal disease, or were immunocompromised.

### 4.3. Outcome Measures

The primary endpoint was the time to achieve complete clinical resolution, which was defined as the full resolution of fever, vomiting, and abdominal pain, along with a decrease in diarrhea frequency to fewer than four episodes per day. Secondary endpoints included the length of hospitalization and the time taken for each individual symptom to resolve.

### 4.4. Statistical Analyses

Continuous variables were reported as means ± standard deviation (SD) or medians (interquartile range, IQR), while categorical variables were presented as counts and percentages. Differences between groups were analyzed using Student’s *t*-test or Wilcoxon’s rank-sum test for continuous variables, and the Chi-square or Fisher’s exact test for categorical variables.

To estimate the time to symptom resolution, Kaplan–Meier survival analysis was employed, with group differences assessed via the log-rank test. Cox proportional hazards regression was used to identify independent predictors of faster recovery, and hazard ratios (HRs) with 95% confidence intervals (CIs) were reported. The proportionality of risks assumption for the Cox models was verified using Schoenfeld residuals (*p* > 0.05). To ensure the stability of the multivariate models, multicollinearity among the baseline variables was assessed using Variance Inflation Factors (VIF). All included covariates demonstrated VIF values below 1.5, indicating the absence of significant collinearity. Statistical significance was set at *p* < 0.05. All analyses were conducted using SAS Version 9.4 (SAS Institute, Cary, NC, USA).

## 5. Conclusions

Early initiation of pathogen-directed macrolide therapy in children with *Campylobacter* enterocolitis results in a statistically significant earlier resolution of clinical symptoms—particularly fever, diarrhea, and abdominal pain—with a median reduction of 2 days in the time to complete clinical resolution, and is associated with a shortened duration of hospitalization by a median of 1 day. These findings offer pediatric-specific evidence that timely, pathogen-directed therapy provides tangible benefits in this common bacterial infection, with reassuring tolerability noted in our cohort.

## Figures and Tables

**Table 1:** Baseline characteristics of patients according to macrolide use.

Variable	Non-Macrolide (*n* = 37)	Macrolide (*n* = 27)	*p*
Male, no (%)	25 (67.6)	16 (59.3)	0.494
Age, years	8 (5, 0)	9 (5, 13)	0.721 *
Duration of illness before treatment, days	2 (1, 3)	1 (1, 2)	0.102
Laboratory parameters at diagnoses			
WBC count (×10^3^/µL)	11,100 (8030–12,580)	8870 (7300–12,960)	0.459
Hb (g/dL)	12.8 (12.4–14.0)	13 (12.3–14.0)	0.905 *
Platelet (×10^3^/µL)	249 (190–271)	240 (215–306)	0.734
CRP (mg/dL)	6.1 (3.5–8.9)	7.8 (3.4–10.8)	0.168 *
BUN (mg/dL)	8.9 (7.9–11.4)	10.2 (8.1–11.6)	0.577
Cr (mg/dL)	0.5 (0.4–0.6)	0.5 (0.4–0.6)	0.519 *
AST (U/L)	25 (22–27)	22 (17–30)	0.330
ALT (U/L)	13 (11–16)	15 (10–21)	0.522
Total bilirubin (mg/dL)	0.4 (0.4–0.6)	0.4 (0.3–0.5)	0.182
Na (mmol/L)	138 (137–139)	136 (134–138)	<0.001 *
K (mmol/L)	4.1 (3.8–4.2)	4.1 (3.9–4.3)	0.188
Cl (mmol/L)	103 (101–104)	101 (100–104)	0.264
Clinical symptoms at presentation			
Fever, no (%)	37 (100)	26 (96.3)	0.422
Diarrhea (≥4 times/day), no (%)	27 (93)	21 (77.8)	0.661
Bloody stool, no (%)	6 (16.2)	7 (25.9)	0.340
Vomiting, no (%)	10 (27)	10 (37)	0.394

Values are presented as numbers (%) for categorical variables and as means ± standard deviation (SD) or medians (interquartile range, IQR) for continuous variables. *p* values are calculated using Chi-square test or Fisher’s exact test for categorical variables and *t*-test * or Wilcoxon rank sum test for continuous variables.

**Table 2:** Comparison of clinical outcomes between macrolide and non-macrolide groups.

Variable	Non-Macrolide(*n* = 37)	Macrolide (*n* = 27)	*p*-Value
Time to complete clinical resolution (days)			<0.001
mean ± SD	3.7 ± 0.8	2.5 ± 0.8	
median (IQR)	4 (3, 4)	2 (2, 3)	
Length of hospitalization (days)			0.002
mean ± SD	4.5 ± 0.9	3.8 ± 0.7	
median (IQR)	4 (4, 5)	4 (4, 4)	
Time to resolution of diarrhea (days)			<0.001
mean ± SD	3.4 ± 0.7	1.8 ± 1.2	
median (IQR)	3 (3, 4)	2 (1, 3)	
Time to resolution of fever (days)			<0.001
mean ± SD	2.8 ± 1.0	1.1 ± 0.6	
median (IQR)	3 (2, 3)	1 (1, 1)	
Time to resolution of vomiting (days)			0.061
mean ± SD	0.2 ± 0.4	0.4 ± 0.5	
median (IQR)	0 (0, 0)	0 (0, 1)	
Time to resolution of abdominal pain (days)			<0.001
mean ± SD	3.1 ± 0.8	2.0 ± 0.6	
median (IQR)	3 (3, 4)	2 (2, 2)	

Values are presented as means ± SD or medians (IQR) as appropriate. Comparisons between groups were performed using *t*-tests or Wilcoxon rank-sum tests for continuous variables.

**Table 3:** Hazard Ratios for time to complete clinical resolution.

Variable	Model 1		Model 2	
	Hazard Ratio (95% CI)	*p*-Value	Hazard Ratio (95% CI)	*p*-Value
Early macrolide use, within 3 days of onset	2.844 (1.654–4.891)	<0.001	2.664 (1.439–4.931)	0.002
Age (years)	1.016 (0.959–1.077)	0.589		
Serum Na at diagnosis (mmol/L)	0.893 (0.806–0.990)	0.031	0.974 (0.868–1.093)	0.657
Serum CRP at diagnosis (mg/dL)	1.048 (0.991–1.109)	0.099		

Hazard ratios were calculated using Cox proportional-hazards regression. Model 1 represents univariate analysis. Model 2 includes covariates with *p* < 0.05 from Model 1 as covariates.

**Table 4:** Hazard ratios for the time to resolution of individual symptoms and the length of hospitalization.

Variable	Model 1		Model 2	
	Hazard Ratio (95% CI)	*p*-Value	Hazard Ratio (95% CI)	*p*-Value
**Length of hospitalization (days)**				
Early Macrolide use, within 3 days of onset	1.694 (1.004–2.858)	0.049	1.694 (1.004–2.858)	0.049
Age (years)	1.016 (0.960–1.076)	0.583		
Serum Na at diagnosis (mmol/L)	0.939 (0.855–1.031)	0.187		
Serum CRP at diagnosis (mg/dL)	1.035 (0.979–1.094)	0.221		
**Time to resolution of diarrhea (days)**				
Early Macrolide use, within 3 days of onset	2.820 (1.664–4.777)	<0.001	2.820 (1.664–4.777)	<0.001
Age (years)	0.992 (0.937–1.051)	0.792		
Serum Na at diagnosis (mmol/L)	0.908 (0.820–1.007)	0.067		
Serum CRP at diagnosis (mg/dL)	1.032 (0.975–1.093)	0.280		
**Time to resolution of fever (Days)**				
Early Macrolide use, within 3 days of onset	4.498 (2.420–8.362)	<0.001	4.498 (2.420–8.362)	<0.001
Age (years)	1.037 (0.974–1.104)	0.251		
Serum Na at diagnosis (mmol/L)	0.927 (0.837–1.027)	0.149		
Serum CRP at diagnosis (mg/dL)	1.034 (0.978–1.094)	0.241		
**Time to resolution of vomiting (days)**				
Early Macrolide use, within 3 days of onset	0.809 (0.489–1.339)	0.409		
Age (years)	0.999 (0.939–1.063)	0.968		
Serum Na at diagnosis (mmol/L)	1.046 (0.944–1.159)	0.392		
Serum CRP at diagnosis (mg/dL)	0.997 (0.947–1.051)	0.921		
**Time to resolution of abdominal pain (Days)**				
Early Macrolide use, within 3 days of onset	2.928 (1.663–5.155)	<0.001	2.843 (1.489–5.428)	0.002
Age (years)	1.044 (0.976–1.116)	0.210		
Serum Na at diagnosis (mmol/L)	0.908 (0.825–0.999)	0.047	0.990 (0.888–1.104)	0.854
Serum CRP at diagnosis (mg/dL)	1.029 (0.975–1.087)	0.298		

Values are presented as means ± SD or medians (IQR) as appropriate. Comparisons between groups were performed using *t*-tests or Wilcoxon rank-sum tests for continuous variables.

## Data Availability

The data presented in this study are available upon request from the corresponding author due to privacy concerns.
